# Senescence-associated hyper-activation to inflammatory stimuli *in vitro*

**DOI:** 10.18632/aging.203396

**Published:** 2021-08-10

**Authors:** Vivekananda Budamagunta, Sahana Manohar-Sindhu, Yang Yang, Yonghan He, Dmitry O. Traktuev, Thomas C. Foster, Daohong Zhou

**Affiliations:** 1 Genetics and Genomics Graduate Program, Genetics Institute, College of Medicine, University of Florida, Gainesville, FL 32610, USA; 2 Department of Pharmacodynamics, College of Pharmacy, University of Florida, Gainesville, FL 32610, USA; 3 Department of Neuroscience, McKnight Brain Institute, College of Medicine, University of Florida, Gainesville, FL 32610, USA; 4 Pharmacology and Therapeutics Graduate Program, Department of Pharmacology and Therapeutics, College of Medicine, University of Florida, Gainesville, FL 32610, USA; 5 Department of Medicine, College of Medicine, University of Florida, Gainesville, FL 32610, USA

**Keywords:** cellular senescence, inflammation, SASP

## Abstract

Aging is associated with an increased susceptibility to adverse inflammatory conditions such as sepsis and cytokine storm. We hypothesized that senescent cells (SnCs) play a central role in this age-associated pathology in part due to their expression of the senescence-associated secretory phenotype (SASP), which may prime SnCs to inflammatory stimulation. To test this hypothesis, we examined the expression of various inflammatory cytokines and chemokines at the levels of gene transcription and protein production in various SnCs *in vitro* in response to lipopolysaccharide (LPS), interleukin-1β (IL1β), and tumor necrosis factor α (TNFα) stimulation. We found that SnCs not only expressed higher basal levels of various inflammatory cytokines and chemokines as a manifestation of the SASP, but more importantly exhibited hyper-activation of the induction of a variety of inflammatory mediators in response to LPS, IL1β and TNFα stimulation as compared with non-SnCs. This senescence-associated hyper-activation is likely mediated in part via the p38MAPK (p38) and NFκB pathways because LPS stimulation elicited significantly higher levels of p38 phosphorylation and NFκB p65 nuclear translation in SnCs when compared to their non-senescent counterparts and inhibition of these pathways with losmapimod (a p38 specific inhibitor) and BMS-345541 (a selective NFκB inhibitor) attenuated LPS-induced expression of *IL6*, *TNFα*, *CCL5*, and *IL1β* mRNA in SnCs. These findings suggest that SnCs may play an important role in the age-related increases in the susceptibility to developing an exacerbated inflammatory response and highlight the potential to use senotherapeutics to ameliorate the severity of various devastating inflammatory conditions in the elderly.

## INTRODUCTION

Advancing age is associated with a multitude of physical and physiological deteriorations that leave the elderly susceptible to a wide variety of pathological conditions [1]. Consequently, there is a steep decline in the health-related quality of life for the elderly [2]. Amongst a wide variety of conditions, increased susceptibility to severe infections (such as COVID-19) and inflammatory conditions (such as sepsis) is one such age-related phenomenon [3–6]. Despite representing under 25% of the population, people older than 60 account for more than 75% of sepsis related deaths [7]. With respect to COVID-19, people over 60 are three times more likely to die from a severe infection than people under 60 [8]. Santesmasses et al., estimated that the risk of dying from COVID-19 doubles with every 6-8 years of increase in chronological age, highlighting the importance of age as a risk factor. In a retrospective study, Chen et al. showed that even with a similar number of comorbidities, patients older than 60 had a significantly higher probability of developing a serious version of COVID-19 compared to younger demographics [9]. The severity of disease progression in these population upon infection is partially attributed to the higher prevalence of severe cytokine storm in the elderly [10, 11]. Though there are many theories as to what makes the elderly susceptible to severe cytokine storm, there is no commonly accepted explanation to this phenomenon. [5, 12].

Cellular senescence is a phenomenon by virtue of which stressed or damaged cells undergo a permanent cell cycle arrest [13, 14]. In healthy individuals, senescent cells (SnCs) are cleared rapidly by the immune system [15]. This clearance mechanism has been shown to become impaired with advancing age, leading to the accumulation of SnCs [16, 17]. In turn, the accumulation of SnCs has been implicated in many age-related pathologies and diseases [18–20]. The detrimental effects of SnCs are partly a consequence of their expression of the senescence-associated secretory phenotype (SASP) [21]. The SASP includes an extensive list of factors such as inflammatory cytokines, chemokines, and matrix metalloproteases (MMPs) [22], which are detrimental to the normal functioning of neighboring cells [23–25]. Hence, we hypothesized that SnCs contribute to the increased severity of infectious diseases and infection-mediated cytokine storm in the elderly through the expression of the SASP. To test this hypothesis, we examined whether SnCs exhibit hyper-activation to LPS, IL1β and TNFα stimulation. Our results show that SnCs indeed have a greater proclivity to become hyper-activated in response to inflammatory insults, resulting in the increased production of a variety of inflammatory cytokines and chemokines when compared to their non-senescent counterparts, which we term senescence-associated hyper-activation. Senescence-associated hyper-activation may be attributable to a higher basal activation of the p38 mitogen activated protein kinase (p38) and NF-κB pathways [26]. These findings lay a foundation to elucidate the important role of SnCs in the age-related increased susceptibility to severe infections and inflammatory conditions.

## RESULTS

### SnCs exhibit a senescence-associated hyper-activation phenotype in response to inflammatory stimulation

**Figure 1:**
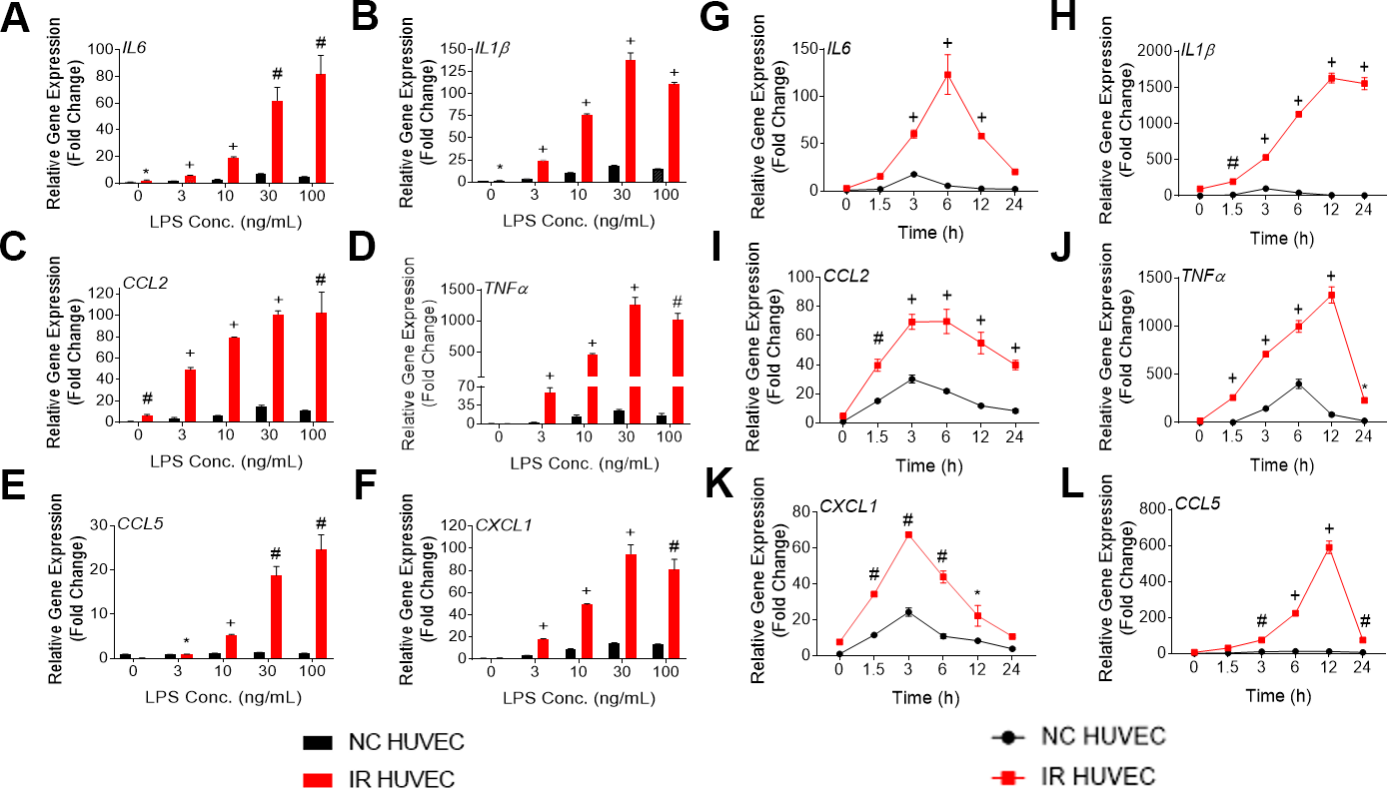
**Lipopolysaccharide (LPS) induces a dose- and time-dependent induction of the senescence-associate secretory phenotype (SASP) gene expression in non-senescent HUVECs (NC HUVEC) and ionizing radiation (IR)-induced senescent HUVECs (IR HUVEC).** (**A**–**F**) Dose response. Relative gene expression of *IL6* (**A**), *IL1β* (**B**), *CCL2* (**C**), *TNFα* (**D**), *CCL5* (**E**), and *CXCL1* (**F**) in NC HUVEC and IR HUVEC stimulated with 3-100 ng/ml LPS for 3 hours. (**G**–**L**), Time course. Relative gene expression of *IL6* (**G**), *IL1β* (**H**), *CCL2* (**I**), *TNFα* (**J**), *CXCL1* (**K**), and *CCL5* (**L**) in NC HUVEC and IR HUVEC as a function of time of stimulation with 30ng/mL LPS. Gene expression in unstimulated NC HUVEC was used as baseline and *GAPDH* was used as endogenous control. (n = 3; mean ± SEM; * p<0.05, # p <0.01, + p<0.001 vs. non-SnC).

While it is well known that SnCs are pro-inflammatory in nature by virtue of expression of the SASP [22, 27], whether inflammatory stimulus could further significantly exacerbate their pro-inflammatory phenotype has not been studied yet. Endothelial cells being a common cell type spread throughout the body in the form of a lining layer of the blood vessels, we decided to use human umbilical vein endothelial cells (HUVEC) to examine this prospect. We also know that HUVEC are inherently responsive to various inflammatory stimuli [28]. To determine if an inflammatory stimulus could significantly exacerbate the pro-inflammatory phenotype of senescent HUVEC compared to their normal counterparts, we examined the transcriptional response of non-senescent (NC HUVEC) and ionizing radiation (IR)-induced senescent HUVEC (IR HUVEC) to lipopolysaccharide (LPS). Using the methods reported by us previously [29], senescence was induced in HUVEC by exposure to ionizing radiation or serial passaging. Induction of senescence in HUVEC by these methods were evidenced by the permanent cell cycle arrest measured by EdU staining (Supplementary Figure 1A), elevated expression of senescence-associated beta galactosidase (SA-β-Gal) activity (Supplementary Figure 1B), increased expression of *CDKN2A* and *CDKN1A* mRNA (Supplementary Figure 1C, 1D) and several SASP factors at basal conditions (Figure 1A–1F and Supplementary Figure 2).

Upon analyzing the transcriptional response of NC HUVEC and IR HUVEC to LPS stimulation, we observed that both types of cells showed a dose-dependent upregulation of mRNA expression for several cytokines and chemokines such as *IL6*, *CCL2*, *CXCL1*, *CCL5*, *IL1β* and *TNFα* (Figure 1A–1F). Moreover, at any given dose, IR HUVEC showed a significantly higher mRNA expression of these inflammatory mediators than their NC HUVEC counterparts (Figure 1A–1F).

Next, we examined the time-dependent dynamics of the mRNA expression of these inflammatory mediators upon LPS stimulation. Much like the dose-dependent response, both NC HUVEC and IR HUVEC exhibited a time-dependent response to LPS stimulation. Again, IR HUVEC expressed significantly higher levels of mRNA for the analyzed cytokines and chemokines, at most given time points, relative to NC HUVEC (Figure 1G–1L).

**Figure 2:**
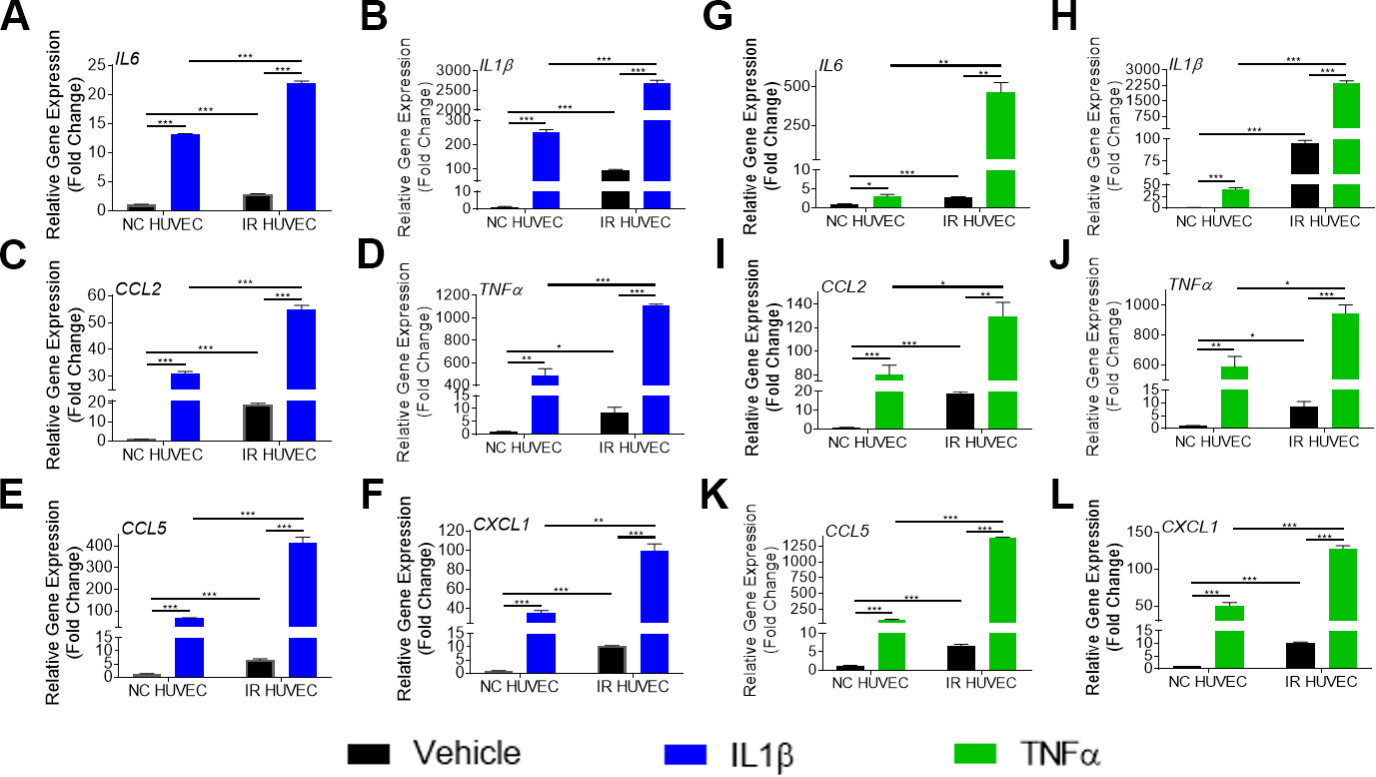
**Comparison of the SASP gene expression in IL1β and TNFα-stimulated NC HUVEC and IR HUVEC.** Relative fold change in gene expression of *IL6* (**A**), *IL1β* (**B**), *CCL2* (**C**), *TNFα* (**D**), *CCL5* (**E**), and *CXCL1* (**F**) in NC HUVEC and IR HUVEC 3 hours after stimulation with 3 ng/mL IL1β. Relative fold change in gene expression of *IL6* (**G**), *IL1β* (**H**), *CCL2* (**I**), *TNFα* (**J**), *CCL5* (**K**), and *CXCL1* (**L**) in NC HUVEC and IR HUVEC 3 hours after stimulation with 3 ng/mL TNFα. Gene expression in unstimulated NC HUVEC was used as baseline and *GAPDH* was used as endogenous control. (n = 3; mean ± SEM; * p<0.05, ** p<0.01, *** p<0.001).

To verify if this exacerbated response of IR HUVEC was specific to LPS, we examined the response of NC and IR HUVEC to IL1β and TNFα, known inflammatory stimulants [28]. As observed with response to LPS, stimulation by IL1β and TNFα elicited a strong transcriptional activation of *IL6*, *CCL2*, *CXCL1*, *CCL5*, *IL1β* and *TNFα* mRNA expression in both NC HUVEC and IR HUVEC. However, stimulated IR HUVEC expressed significantly higher levels of mRNA for all tested cytokines and chemokines when compared to their NC counterparts (Figure 2A–2L). These results suggest that IR HUVEC are hyper-reactive to inflammatory stimulus, which we term senescence-associated hyper-activation.

To examine if this phenomenon was exclusive for IR HUVEC, we generated replicative senescent HUVEC (Rep-Sen HUVEC) and tested their response to LPS, IL1β and TNFα. Rep-Sen HUVEC, similar to IR HUVEC, showed exacerbated transcriptional activation of *IL6, CXCL10* and *CCL5* upon inflammatory stimulation (Supplementary Figure 2A–2I).

To explore whether senescence-associated hyper-activation is a general characteristic of SnCs, rather than being specific to endothelial cells, studies were extended to IR-induced senescent human adipose derived stem cells (ASCs) (Supplementary Figure 3A–3I), renal epithelial cells (RECs) (Supplementary Figure 4A–4I) and WI38 lung fibroblast (WI38) (Supplementary Figure 5A–5I). SnCs from all three cell types exhibited a higher basal level of *IL6*, *CCL5* and *CXCL10* mRNA expression as well as higher expression of these inflammatory mediators in response to LPS, IL1β and TNFα stimulation than non-SnCs with a few exceptions in which some of the cells were not very responsive to LPS stimulation. For example, non-senescent WI38 fibroblasts showed no significant change in expression upon LPS stimulation for any of the three genes analyzed, whereas senescent WI38 fibroblasts showed a significant upregulation of mRNA for CCL5, but not for IL6 and CXCL1 upon LPS stimulation.

Cumulatively, this data suggests that SnCs exhibit a senescence-associated hyper-activation phenotype upon being stimulated with a prominent inflammatory stimulant.

### SnCs secrete high levels of inflammatory cytokines and chemokines

**Figure 3:**
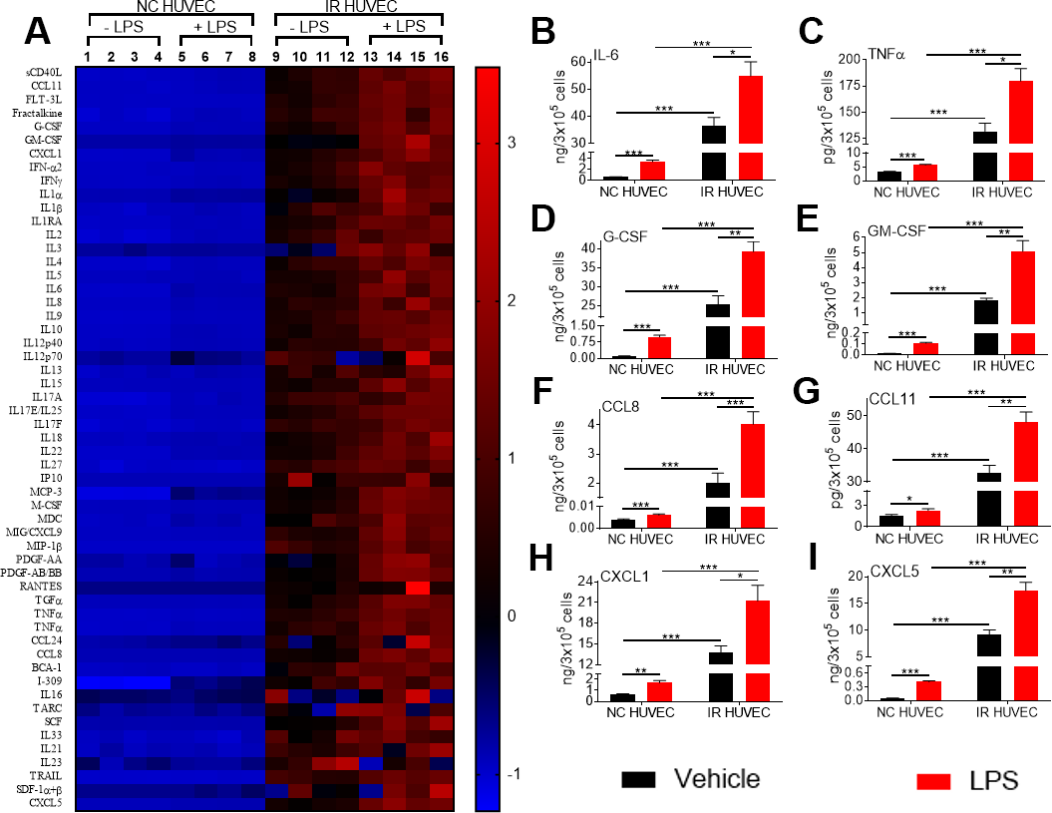
**Comparison of the production of inflammatory cytokines and chemokines by NC HUVEC and IR HUVEC.** (**A**) Heat-map representing the normalized concentrations of inflammatory cytokines and chemokines in the conditioned media of NC HUVEC and IR HUVEC stimulated with vehicle or LPS (30 ng/ml) for 24 hours. (**B**–**I**) Normalized concentration of IL6 (**B**), TNFα (**C**), G-CSF (**D**), GM-CSF (**E**), CCL8 (**F**), CCL11 (**G**), CXCL1 (**H**), and CXCL5 (**I**), produced by NC HUVEC and IR HUVEC stimulated with vehicle or LPS (30 ng/ml) for 24 hours. (n = 4; mean ± SEM; * p<0.05, ** p<0.01, *** p<0.001).

To investigate whether the increased mRNA levels for the multiple inflammatory mediators in SnCs translate into an elevated secretion of the corresponding factors, we analyzed the conditioned media from NC HUVEC and IR HUVEC with or without LPS stimulation. From the data presented in Figure 3 and Supplementary Table 1, it is evident that IR HUVEC secreted significantly higher levels of multiple cytokines and chemokines relative to NC HUVEC under basal condition. More importantly, when stimulated with LPS, IR HUVEC produced much greater levels of these factors than LPS-stimulated NC HUVEC (Figure 3 and Supplementary Table 1), confirming that SnCs indeed have a senescence-associated hyper-activation phenotype.

### Senescence-associated hyper-activation is not associated with increased expression of the Toll-like receptor4 (TLR4)

**Figure 4:**
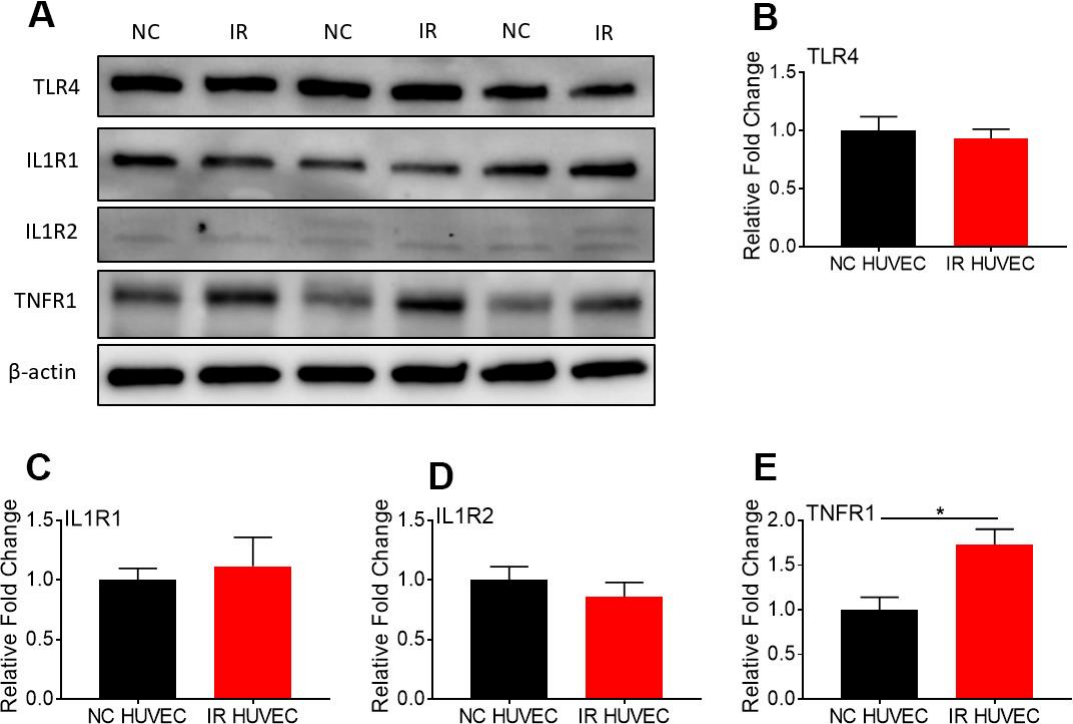
**Comparison of the expression of surface receptors of inflammatory stimulants in NC HUVEC and IR HUVEC.** Representative western-blot images (**A**) and densitometry based quantitative analysis of TLR4 (**B**) IL1R1 (**C**), IL1R2 (**D**), and TNFR1 (**E**) in NC HUVEC and IR HUVEC. β-actin was used as a loading control. (n = 3; mean ± SEM; * p<0.05).

To investigate whether senescence-associated hyper-activation is due to an increased expression of surface receptors for the inflammatory stimulants, we quantified the expression of TLR4 (a primary receptor for LPS [30]), IL1 receptor 1 (IL1R1), IL1R2 and TNF receptor 1 (TNFR1) in NC and IR HUVECs using western blotting (Figure 4). We found that both cells expressed similar levels of TLR4 (Figure 4B), IL1R1 (Figure 4C) and IL1R2 (Figure 4D), while IR HUVECs expressed a slightly higher level of TNFR1 than NC HUVECs (Figure 4E). This finding suggests that the senescence-associated hyper-activation in responses to LPS and IL1β stimulation is not due to an increased expression of surface receptors but possibly due to the changes in their downstream pathways. However, increased expression of TNFR1 may partially contribute to the senescence-associated hyper-activation to TNFα.

### Senescence-associated hyper-activation is mediated by the activation of p38

**Figure 5:**
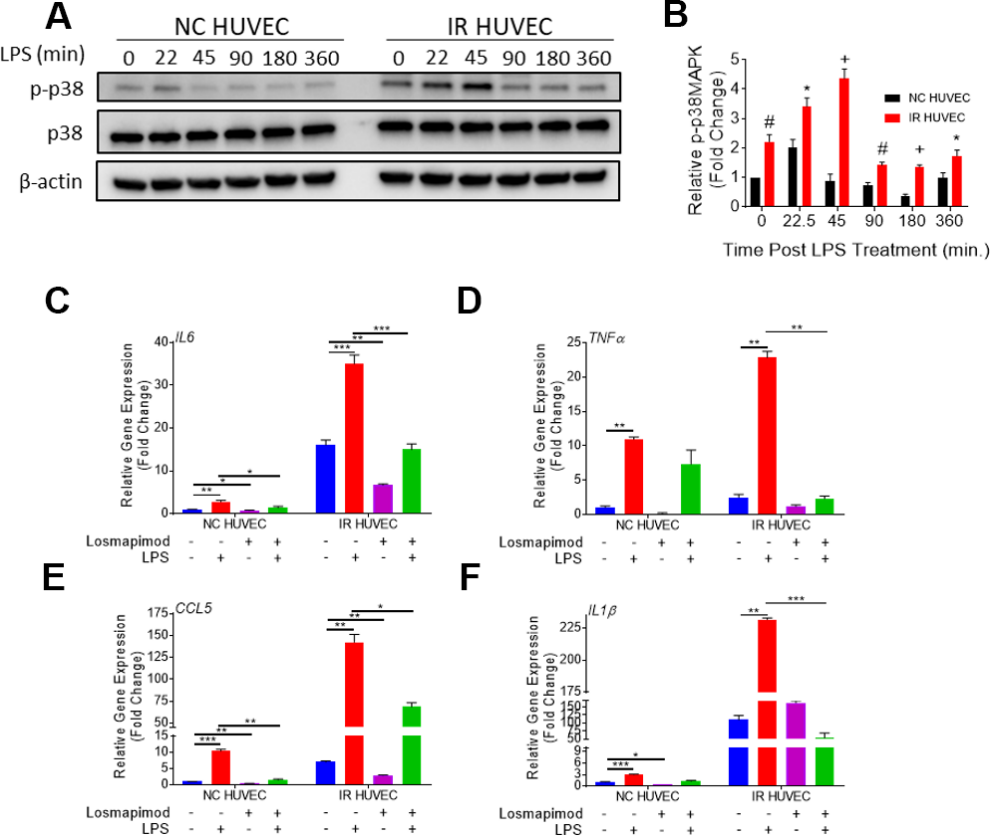
**Regulation of senescence-associated hyper-activation via p38-MAPK (p38) pathway.** (**A**, **B**) IR HUVEC exhibit higher activation of p38 than NC HUVEC. Representative western-blot images (**A**) and densitometry based quantitative analysis (**B**) of phosphorylated p38 (p-p38) and total p38 (p38) in NC HUVEC and IR HUVEC stimulated with LPS (30 ng/ml) for 0-6 hours. (n = 3; mean ± SEM; * p<0.05, # p <0.01, + p<0.001 vs. NC HUVEC). β-actin was used as a loading control. (**C**–**F**) p38 inhibition attenuates the expression of *IL6* (**C**), *TNFα* (**D**), *CCL5* (**E**), and *IL1β* (**F**) mRNA in IR-HUVEC. NC HUVEC and IR-HUVEC were exposed to LPS (30 ng/ml) or the p38 inhibitor losmapimod (1 μM) or their combination for 3 hours followed by mRNA analysis. Gene expression in unstimulated NC HUVEC was used as baseline and *GAPDH* was used as endogenous control (n = 3; mean ± SEM; * p<0.05, ** p<0.01, *** p<0.001).

We hypothesized that senescence-associated hyper-activation is attributable to the hyper-activity of some of the intracellular signaling pathways responsible for the SASP phenotype. To test this hypothesis, we examined if the p38 pathway becomes hyper-activated in SnCs upon inflammatory stimulation. Our results not only corroborated previous demonstrations of SnCs having an elevated basal p38 activation [26], but also showed that IR HUVEC exhibited a significantly higher activation of p38 upon IPS stimulation compared to NC HUVEC (Figure 5A, 5B).

To determine whether senescence-associated hyper-activation effect was dependent on the activation of p38, NC HUVEC and IR HUVEC were pre-treated with losmapimod, a specific p38 inhibitor [31], prior to LPS stimulation. Our analysis showed that the upregulation of *IL6*, *TNFα*, *CCL5,* and *IL1β* mRNA expressions in IR HUVEC with or with LIP stimulation were abrogated or significantly reduced by the losmapimod pretreatment, demonstrating that activation of p38 mediates not only the expression of SASP at the basal conditions but also that of senescence-associated hyper-activation in response to LPS stimulation (Figure 5C–5F).

### Senescence-associated hyper-activation is also mediated by the activation of the NF-κB pathway

**Figure 6:**
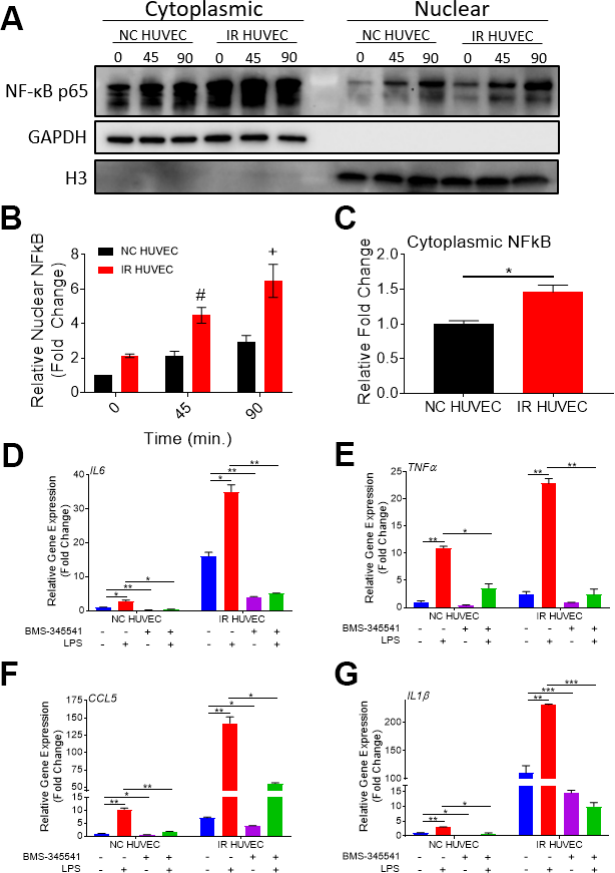
**Regulation of senescence-associated hyper-activation via NF-κB pathway.** (**A**–**C**) IR HUVEC exhibit higher baseline expression and activation of NF-κB when compared to NC HUVEC. Representative western-blot images (**A**) (the middle line is the molecular weight markers), densitometry based quantitative analysis of nuclear fraction (**B**) and cytoplasmic fraction (**C**) of NF-κB p65 in NC HUVEC and IR HUVEC stimulated with LPS (30 ng/ml) for 0-90 min (n = 3; mean ± SEM; * p<0.05, # p <0.01, + p<0.001 vs. NC HUVEC). Histone H3 and GAPDH were used as the loading control for nuclear and cytoplasmic proteins, respectively. (**D**–**G**) NF-κB inhibition attenuates the expression of *IL6* (**D**), *TNFα* (**E**), *CCL5* (**F**), and *IL1β* (**G**) mRNA in IR HUVEC. NC HUVEC and IR HUVEC were treated with LPS (30 ng/ml) or the NF-κB inhibitor BMS-345541 (10 μM) or their combination for three hours followed by mRNA analysis. Gene expression in unstimulated NC HUVEC was used as baseline and *GAPDH* was used as endogenous control. (n = 3; mean ± SEM; * p<0.05, ** p<0.01, *** p<0.001).

Based on the study by Freund et al., showing that p38-MAPK acts upstream of the NF-κB pathway to induce SASP, we hypothesized that NF-κB activation also mediates senescence-associated hyper-activation. To test this hypothesis, we analyzed NF-κB p65 levels in the cytoplasm and nucleus of NC HUVEC and IR HUVEC at baseline and upon stimulation with LPS, IL1β and TNFα, by immunocytochemistry (Supplementary Figure 6) and by immunoblotting (Figure 6A, 6B). Both results showed that IR HUVEC exhibited a significantly higher baseline level of nuclear NF-κB, as well as a significantly higher nuclear translocation of NF-κB upon LPS stimulation than NC HUVEC (Figure 6A, 6B and Supplementary Figure 6). Similarly, IR HUVEC showed a greater translocation of NF-κB p65 into the nucleus upon stimulation with IL1β and TNFα than NC HUVEC (Supplementary Figure 6). Collectively, these results suggest that IR HUVEC respond to inflammatory stimulation by hyper-activation of the NF-κB pathway.

To validate the dependence of senescence-associated hyper-activation on NF-κB activity, NC HUVEC and IR HUVEC were treated with BMS-345541, a potent inhibitor of NF-κB activation [32], prior to LPS stimulation. Our analysis revealed that the upregulation of *IL6*, *TNFα*, *CCL5* and *IL1β* mRNA expressions in IR HUVEC with or without LPS stimulation were abrogated or significantly reduced by the pretreatment with BMS-345541 (Figure 6C–6F), confirming that the NF-κB pathway plays an important role in the induction of both SASP and senescence-associated hyper-activation in IR HUVEC.

## DISCUSSION

An increased proclivity to severe infections and infection-induced adverse cytokine storm can be observed in the aging population [3, 5, 8, 11, 12]. Here we report the discovery of a novel phenomenon named senescence-associated hyper-activation in SnCs and demonstrated that common inflammatory stimuli such as LPS, IL1β and TNFα, which are relevant to many infections and inflammatory conditions, induce hyper-activation in SnCs. This senescence-associated hyper-activation makes SnCs produce a large amount of various inflammatory cytokines and chemokines including IL6, a cytokine believed to play a central role in the development of a cytokine storm [33, 34]. These findings are in agreement with the prior observation by Chambers et al., that skin biopsies from both young and old patients showed similar baseline expression of chemokines, such as *CXCL1*, *CCL2* and *CCL8* but skin biopsies from older population had a significantly higher expression of these chemokines under inflammatory conditions [6]. Additionally, Saito et al. also reported that old mice subjected to LPS injection or cecal ligation puncture (CLP) expressed significantly higher levels of *IL6* and *TNFα* mRNA in lungs and hearts than the tissues from young mice [3].

Extrapolating from previously published studies, senescence-associated hyper-activation may be an underlying cause of the high susceptibility to infection-induced severe inflammation and cytokine storm in older populations. For example, IR HUVEC showed a significantly higher production of various inflammatory cytokines as IL6, G-CSF, and TNFα (Figure 3B–3I), known markers of a poor prognosis in severe infectious conditions including COVID-19 [35–37]. We speculate that senescence burden and senescence-associated hyper-activation may contribute to the severe cases of COVID-19 that disproportionately impact the elderly [5]. In addition, these findings could partly explain the findings by Saito et al., showing that while young and old mice had a similar baseline of serum cytokines, an inflammatory insult with LPS triggered the generation of significantly higher quantities of these cytokines in older mice compared to their younger counterparts [3]. Therefore, further studies are needed to determine if accumulation of SnCs is responsible for developing high susceptibility to severe cytokine storm seen in the elderly under various pathological conditions.

Our study also revealed that senescence-associated hyper-activation is not necessarily an outcome of an altered expression of surface receptors of the inflammatory stimulants but more probably attributable to the changes in the downstream pathways such as the p38 and NF-κB signaling pathways as reported previously [26]. As both these pathways have been shown to be crucial for the expression of the SASP [26, 38], it is likely that SnCs, by virtue of their SASP regulating networks, are primed to react more aggressively to inflammatory stimuli when compared to their normal counterparts despite not having an increased expression of receptors upstream of these pathways. The involvement of the p38 pathway in senescence-associated hyper-activation is in agreement with a report from Vukmanovic-Stejic et al., which shows that the enhanced cutaneous inflammation seen in the elderly injected with varicella zoster virus antigen, was attenuated by treatment with losmapimod, a highly specific p38 inhibitor [39]. Though the dysregulated cutaneous inflammation was initially not linked to cellular senescence, a recent study by Chambers et al., demonstrated the involvement of cellular senescence in this phenomenon, making it relevant to our study [6]. In addition, these results also provide additional evidence as to why p38 inhibition is a promising therapeutic option for the treatment of severe COVID-19 patients [40].

In conclusion, we discovered that SnCs exhibit hyper-activation upon an inflammatory insult, which we termed senescence-associated hyper-activation. Our results suggest that SnCs could contribute to the age-related predisposition of the body to develop stronger cytokine storm upon infections. This calls for a paradigm shifting study from considering SnCs as indirect participants in inflammatory pathologies to being recognized as central players in these processes. Discovering the senescence-associated hyper-activation phenomenon also highlights an opportunity and the urgent need for testing the possibilities that the newly developed senotherapeutics may have the potential to mitigate the incidence of life-threatening inflammatory conditions in the elderly and potentially lengthen their health-span.

## MATERIALS AND METHODS

### Cell culture

Human umbilical vein endothelial cells (HUVEC, Cat. No. PCS-100-010), human WI38 fibroblasts (WI38, Cat. No. CCL-75) and human renal epithelial cells (REC, Cat. No. PCS-400-012) were obtained from the American Type Culture Collection (ATCC, Manassas, VA, USA). Adipose stem/stromal cells (ASC) were previously isolated from adipose tissue (female donors) obtained during elective liposuction procedure (Traktuev et al., 2008). HUVEC were cultured in EBM-2 (Cat. No. CC-3156, Lonza, Basel, Switzerland) media supplemented with EGM-2 SingleQuots (Cat. No. CC-4176, Lonza, Basel, Switzerland). WI38 cells were cultured in DMEM medium (Cat. No. 12430054, Thermo Fisher Scientific, Waltham, MA, USA) supplemented with 10% fetal bovine serum (FBS, Cat. No. 89510-188, VWR, Radnor, PA, USA). REC were cultured in REBM medium (CC-3191, Lonza, Basel, Switzerland) supplemented with REGM SingleQuots (CC-4127, Lonza, Basel, Switzerland). ASC were cultured in EBM-2 (Cat. No. CC-3156, Lonza, Basel, Switzerland) media supplemented with EGM-2MV SingleQuots (Cat. No. CC-4147, Lonza, Basel, Switzerland). To prevent contamination all culture media were supplemented with 100 U/mL penicillin and 100 μg/mL streptomycin (Cat. No. 15140122, Thermo Fisher Scientific, Waltham, MA, USA). Cells were cultured in a humidified incubator at 37° C with 5% CO_2_.

### Senescence induction

Cells of early passages (HUVEC < 10 passages; WI-38 < 25 passages; REC < 10 passages; and ASC < 4 passages) were considered to be non-senescent cells (NC). Two methods of senescence induction - replicative exhaustion and ionizing radiation, were used as previously described [29]. In short, to generate replicative senescent cells (SnCs), HUVEC were passaged until they stopped replicating further. To generate IR-induced SnCs, all cell types were exposed to 20 Gy of X-rays, followed by a culturing for 10 days. The induction of cellular senescence was validated by analyzing the expression of *CDKN2A* and *CDKN1A* mRNA, positive of SA-β-Gal, and lack of cell proliferation. SA-β-Gal was tested using SA-β-Gal staining kit (Cat. No. 9860, Cell Signaling Technologies, Danvers, MA, USA), and cell proliferation was assessed using EdU cell proliferation kit (Cat. No. C10337, Invitrogen, Carlsbad, CA, USA), following the manufacturers’ instructions.

### Inflammatory stimulation

Various concentrations of LPS (Cat. No. L4391, Sigma-Aldrich, St. Louis, MO, USA), IL1β (Cat. No. 200-01B, Peprotech, Rocky Hill, NJ, USA), and TNFα (Cat. No. 300-01A, Peprotech, Rocky Hill, NJ, USA) were used to stimulate cells for different durations of time at the indicated concentrations and times presented in each figure legend.

### Analysis of the production of various inflammatory cytokines and chemokines

NC HUVEC and IR HUVEC were cultured with medium alone or with 30 ng/mL LPS for 24 hours. The conditioned media from the cultures was then harvested and subjected to analysis using Human Cytokine Array/Chemokine Array 71-Plex Panel by the Eve Technologies Corporation (Calgary, AB, Canada). Values are listed in Supplementary Table 1 (Supplementary Table 1).

### Quantitative polymerase chain reaction (qPCR)

RNA isolated from cells using RNeasy mini kit (Cat. No. 74106, Qiagen, Hilden, Germany) was converted into cDNA using a high capacity cDNA reverse transcription kit (Cat. No. 4368813, Applied Biosystems, Foster City, CA, USA). Gene expression was then quantified using gene specific primers (Supplementary Table 2) and fast SYBR green master-mix (Cat. No. 4385617, Applied Biosystems. Foster City, CA, USA) as per the manufacturer’s instructions. The expression of *GAPDH* was used for normalization. Level of gene expression in untreated NC cells was used as baseline and fold change in gene expression was defined based on ΔΔCT method.

### Pathway inhibitory studies

NC HUVEC and IR HUVEC were pre-treated with 1μM of losmapimod (Cat. No. HY10402, Med Chem Express, New Jersey, USA) or BMS-345541 (Cat. No. HY-10519, Med Chem Express, New Jersey, USA) for 2 hours before the cells were stimulated with 30 ng/mL LPS for 3 hours before being harvested for RNA isolation for qPCR.

### Whole protein isolation and sample preparation for immunoblot analysis

Control and treated cells were washed with 1X PBS before RIPA buffer (Cat. No. BP-115, Boston Bioproducts, Ashland, MA, USA) was added to the dish. The resultant lysate was then frozen at -80° C for later use. These protein lysates were then thawed and quantified using Pierce BCA protein assay kit (Cat. No. 23225, Thermo Fisher Scientific, Waltham, MA, USA) and normalized before adding 4X Laemmli loading buffer (Cat. No. BP-110R, Boston Bioproducts, Ashland, MA, USA). Samples were then heated to 95° C for 10 minutes before using for western blotting as we previously reported [29].

### Cell fractionation and protein isolation

Cells were trypsinised and harvested as pellets after various treatments. They were lysed and then fractionated to obtain cytoplasmic and nuclear proteins for immunoblot analysis using the NE-PER nuclear and cytoplasmic extraction kit (Cat. No. 78833, Thermo Fisher Scientific, Waltham, MA, USA) following the manufacturer’s instructions.

### Western blotting

Western blotting was done as previously described [29]. In short, 10 μL of each prepared protein sample was used to run western blot using 15-well 4-20% Mini-PROTEAN TGX Precast Protein Gels (Cat. No. 4561096, Bio-Rad Laboratories, Hercules, CA, USA). The samples were run at 200V for 45 minutes before transferring onto a PVDF membrane using the Trans-Blot turbo transfer system (Bio-Rad Laboratories, Hercules, CA, USA). The PVDF membranes were blocked with 5% milk for 1 hour at room temperature before incubating with primary antibody overnight at 4° C. The membranes were then washed and incubated with horse radish peroxidase (HRP)-conjugated secondary antibody for 1 hour at room temperature. Protein-antibody complexes were revealed with HRP substrate on ChemiDoc imaging system (Bio-Rad Laboratories, Hercules, CA, USA). Antibodies used are listed in Supplementary Table 3 (Supplementary Table 3).

### Statistical analysis

Data are expressed as mean ± *SEM*, unless mentioned otherwise. When comparing more than two groups, data sets were analyzed using repeated measures analysis of variance (ANOVA) on Graphpad Prism (San Diego, CA, USA). Post hoc comparisons were performed between group means using Sidak multiple comparisons test*.* To compare two groups, Student's *t* test was used. *p* < 0.05 was considered significant.

## Supplementary Material

Supplementary Figures

Supplementary Tables
