# Supplementary material for: Senescence-associated hyper-activation to inflammatory stimuli in vitro
Source: Aging (Albany NY). 2021 Aug 10;13(15):19088–107. doi: 10.18632/aging.203396 (PMC8386536; doi:10.18632/aging.203396)
Supplement: Supplementary Figures [file aging-13-203396-s001.pdf]

## SUPPLEMENTARY FIGURES

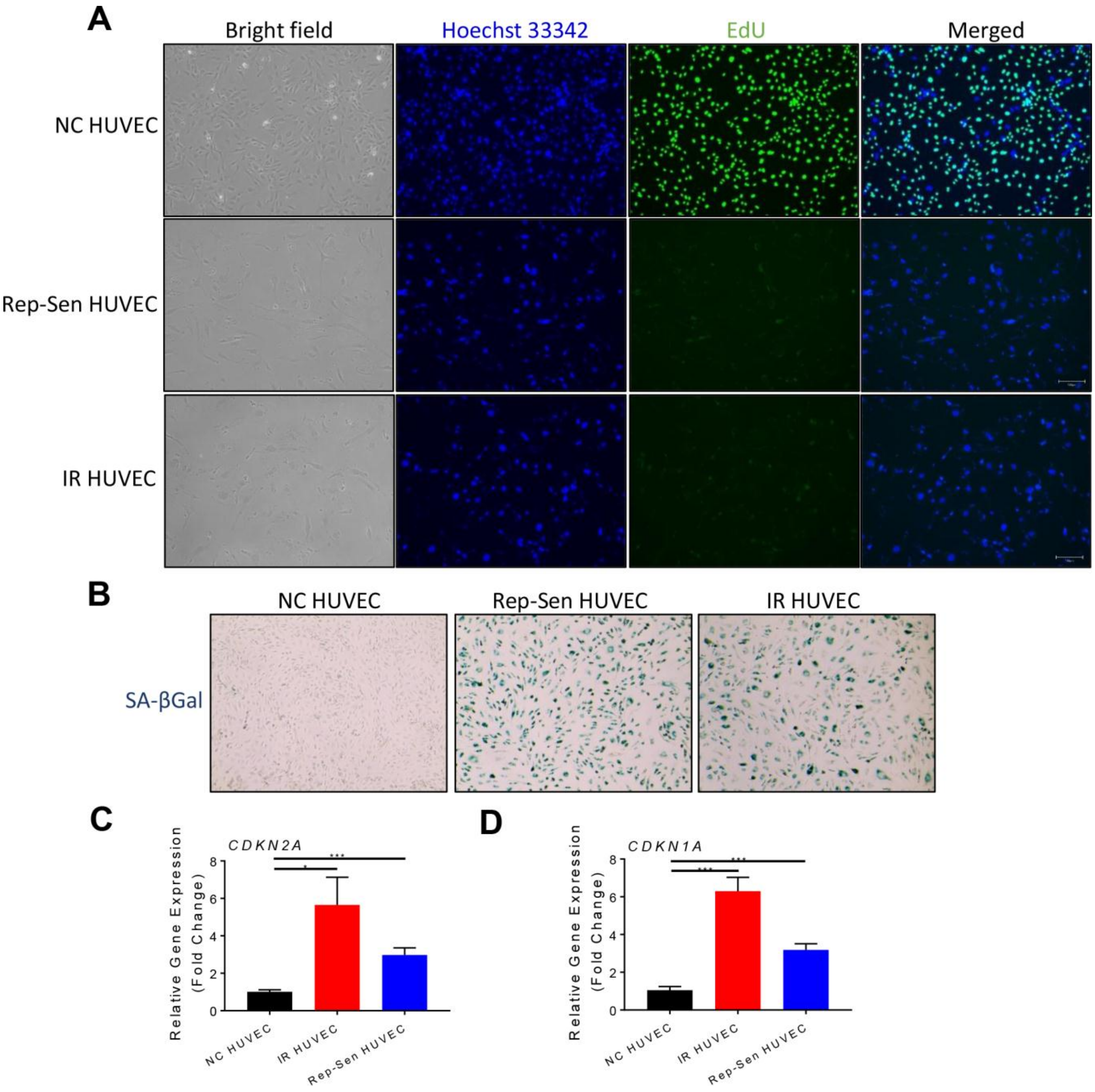


**Supplementary Figure 1. Validation of HUVEC senescence.** (**A**) Representative images of non-senescent (NC HUVEC), IR-induced senescent HUVECs (IR HUVEC) and replicative senescent HUVECs (Rep-Sen HUVEC) stained for EdU to analyze cell cycle arrest. Nuclei were revealed with Hoechst 33342 staining. (**B**) Representative images of NC HUVEC, IR HUVEC and Rep-Sen HUVEC stained for senescence associated β-galactosidase (SA-β-Gal; blue color). (**C**, **D**) Relative mRNA expression of *CDKN2A* (**C**) and *CDKN1A* (**D**) in NC HUVEC, IR HUVEC and Rep-Sen HUVEC. Gene expression in unstimulated NC HUVEC was used as baseline and *GAPDH* was used as endogenous control (n = 3; mean ± SEM; * p<0.05, *** p<0.001).

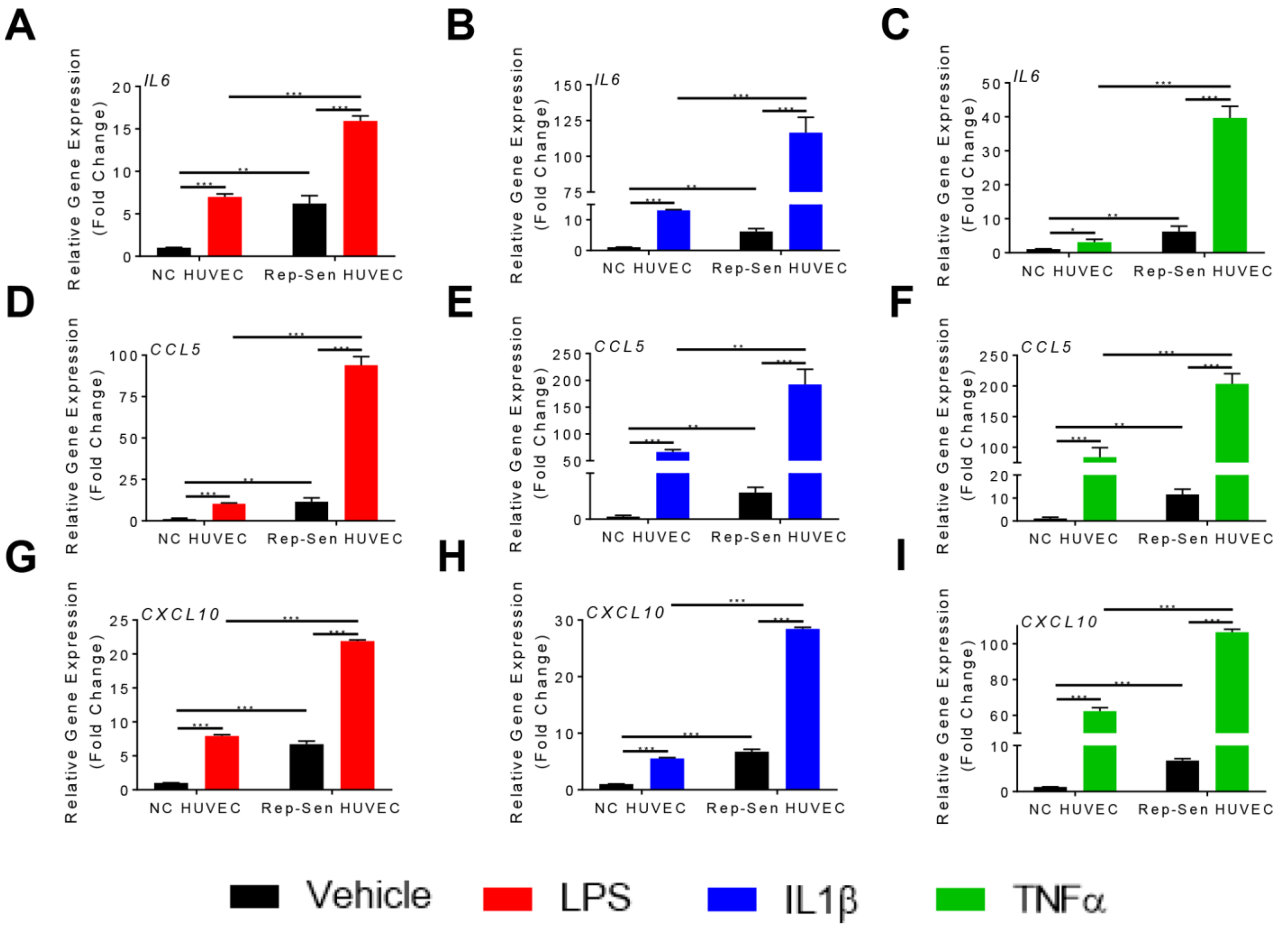


**Supplementary Figure 2. Transcriptional response of NC HUVEC and Rep-Sen HUVEC to LPS, IL1β and TNFα stimulation.** Relative change in mRNA expression of *IL6* (**A**–**C**), *CCL5* (**D**–**F**), and *CXCL10* (**G**–**I**) in NC HUVEC and Rep-Sen HUVEC stimulated with 30 ng/mL LPS (**A**, **D**, **F**), 3 ng/ml IL1β (**B**, **E**, **H**), and 3 ng/ml TNFα (**C**, **F**, **I**) for 3 hours. Gene expression in unstimulated NC HUVEC was used as baseline and *GAPDH* was used as endogenous control (n = 3; mean ± SEM; * $p<0.05$, ** $p<0.01$, *** $p<0.001$).

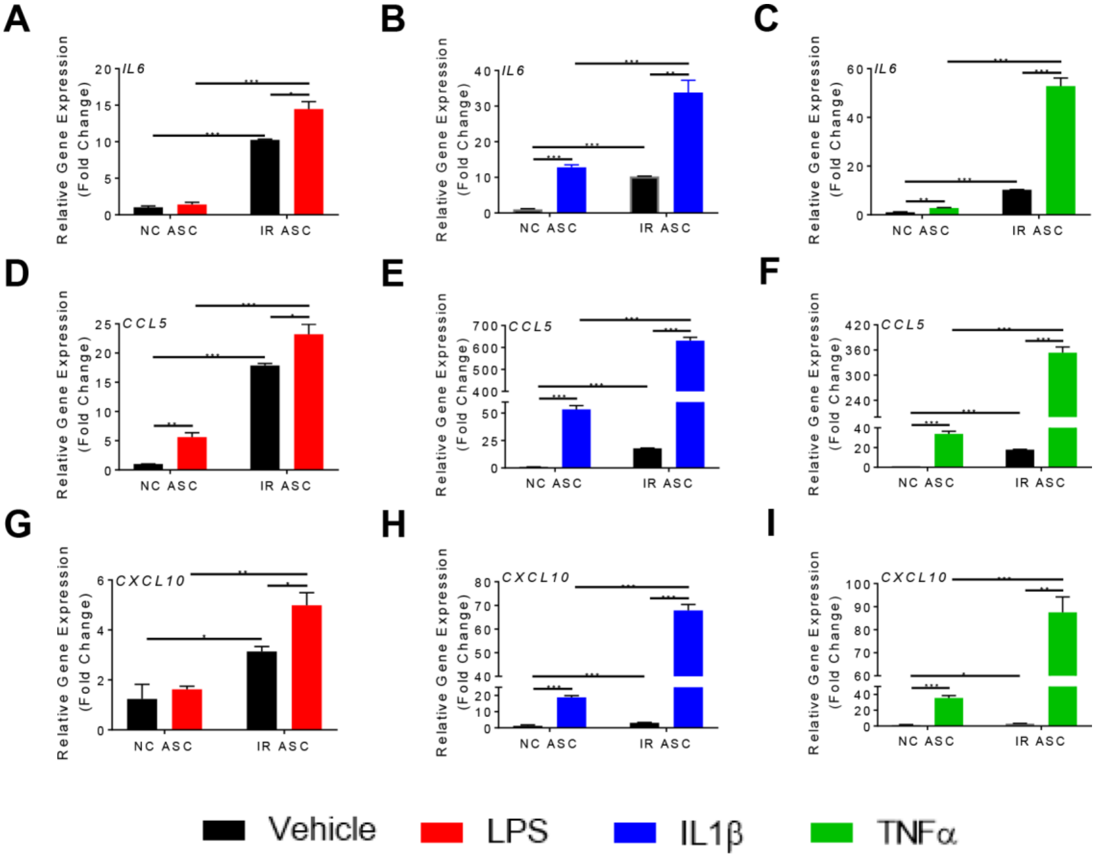


**Supplementary Figure 3. Transcriptional response of non-senescent adipose stem/stromal cells (NC ASC) and IR-induced senescent ASC (IR ASC) to LPS, IL1β and TNFα stimulation.** Relative change in mRNA expression of *IL6* (**A**–**C**), *CCL5* (**D**–**F**), and *CXCL10* (**G**–**I**) in NC ASC and IR ASC stimulated with 30 ng/mL LPS (**A**, **D**, **F**), 3 ng/ml IL1β (**B**, **E**, **H**), and 3 ng/ml TNFα (**C**, **F**, **I**) for 3 hours. Gene expression in unstimulated NC ASC was used as baseline and *GAPDH* was used as endogenous control (n = 3; mean ± SEM; * p<0.05, ** p<0.01, *** p<0.001).

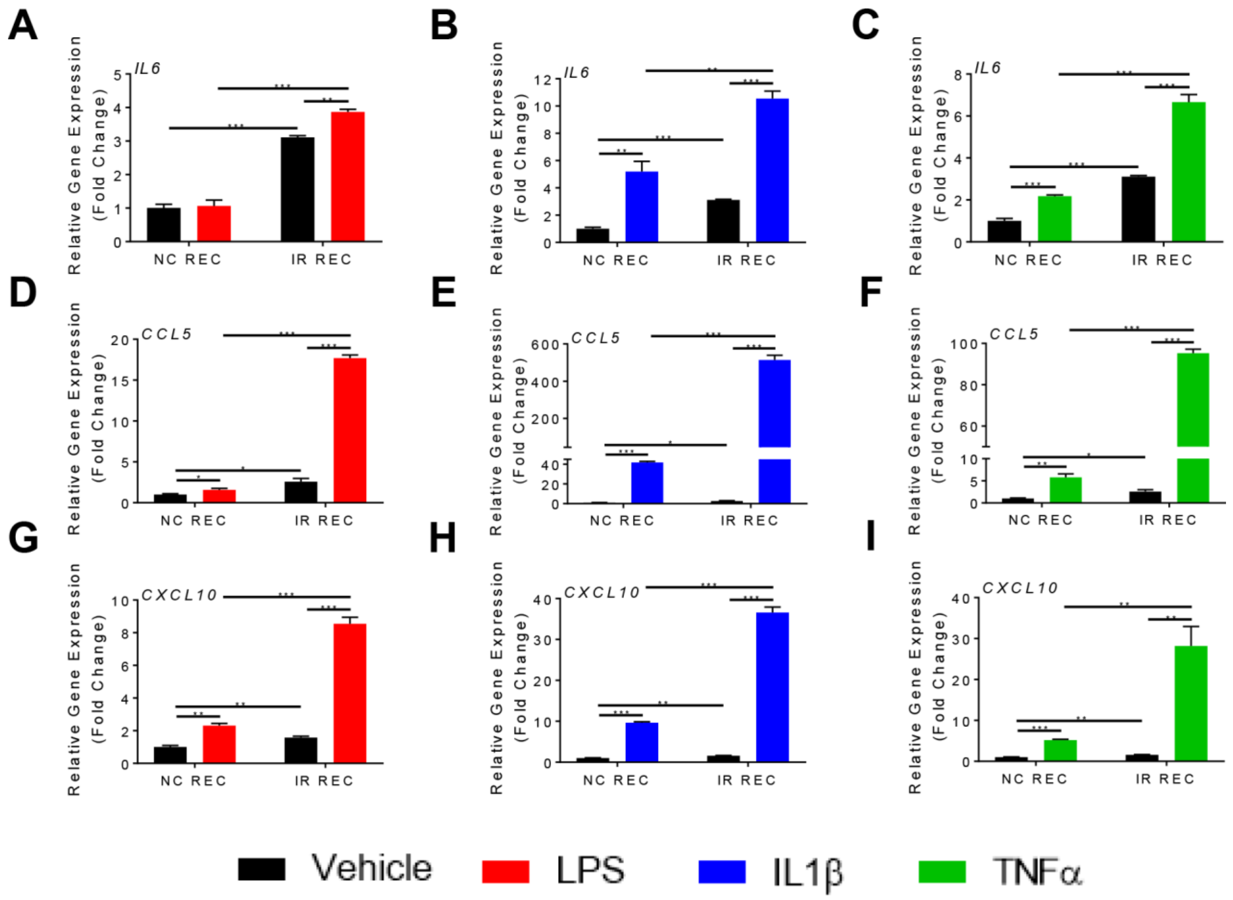


**Supplementary Figure 4. Transcriptional response of non-senescent human renal epithelial cells (NC REC) and IR-induced senescent RECs (IR REC) to LPS, IL1β and TNFα stimulation.** Relative change in mRNA expression of *IL6* (**A**–**C**), *CCL5* (**D**–**F**), and *CXCL10* (**G**–**I**) in NC REC and IR REC stimulated with 30 ng/mL LPS (**A**, **D**, **F**), 3 ng/ml IL1β (**B**, **E**, **H**), and 3 ng/ml TNFα (**C**, **F**, **I**) for 3 hours. Gene expression in unstimulated NC REC was used as baseline and *GAPDH* was used as endogenous control (n = 3; mean ± SEM; * p<0.05, ** p<0.01, *** p<0.001).

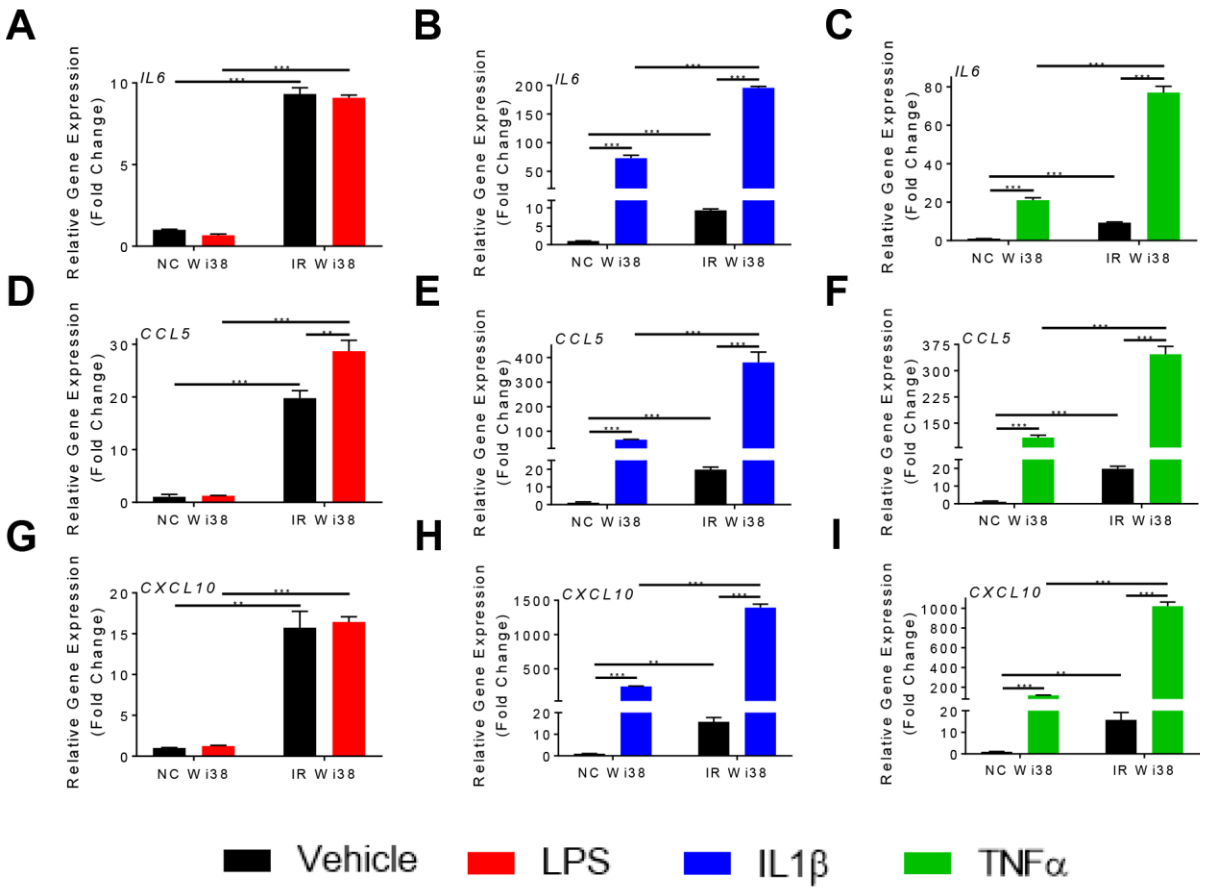


**Supplementary Figure 5. Transcriptional response of non-senescent human WI38 lung fibroblast cells (NC WI38) and IR-induced senescent WI38 cells (IR WI38) to LPS, IL1β and TNFα stimulation.** Relative change in mRNA expression of *IL6* (**A**–**C**), *CCL5* (**D**–**F**), and *CXCL10* (**G**–**I**) in NC WI38 and IR WI38 stimulated with 30 ng/mL LPS (**A**, **D**, **F**) 3 ng/ml IL1β (**B**, **E**, **H**), and 3 ng/ml TNFα (**C**, **F**, **I**) for 3 hours. Gene expression in unstimulated NC WI38 was used as baseline and *GAPDH* was used as endogenous control (n = 3; mean ± SEM; * p<0.05, ** p<0.01, *** p<0.001).

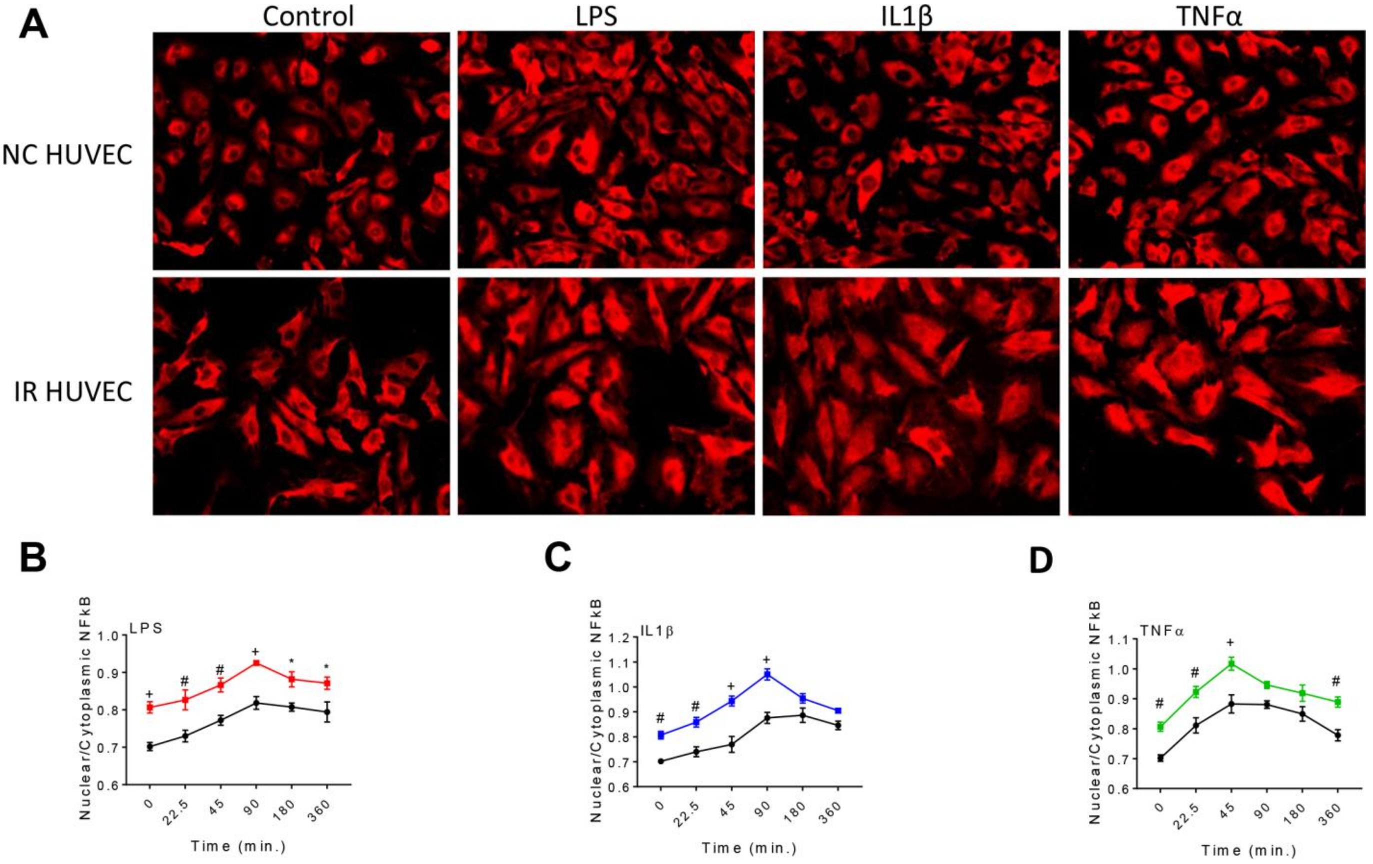


**Supplementary Figure 6. Assessment of NF-κB nuclear translocation in HUVEC in response to LPS, IL1β and TNFα stimulation.** (**A**) Representative images of NF-κB p65 immunostaining in NC HUVEC and IR HUVEC cultured with vehicle (Control) or stimulated with LPS (30 ng/ml), IL1β (3 ng/ml) or TNFα (3 ng/ml) for 90 min. (**B**–**D**) Quantitative analysis of the nuclear/cytoplasmic ratio of NF-κB p65 immunostaining in NC HUVEC and IR HUVEC stimulated with 30 ng/ml LPS (**B**), 3 ng/ml IL1β (**C**) or 3 ng/ml TNFα (**D**) at various time points (n = 4; mean ± SEM; * $p<0.05$, ** $p<0.01$, *** $p<0.001$).
