# Supplementary material for: Senescence-associated hyper-activation to inflammatory stimuli in vitro
Source: Aging (Albany NY). 2021 Aug 10;13(15):19088–107. doi: 10.18632/aging.203396 (PMC8386536; doi:10.18632/aging.203396)
Supplement: Supplementary Tables [file aging-13-203396-s002.pdf]

SUPPLEMENTARY TABLES

Supplementary Table 1. Production of inflammatory cytokines and chemokines by non-senescent HUVECs (NC HUVEC) and IR-induced senescent HUVECs (IR HUVEC) 24 hours after incubation with vehicle or stimulated with 30 ng/ml LPS.

	1	2	3	4	P-Value			
	NC HUVEC + vehicle (pg/mL)	NC HUVEC + LPS (pg/mL)	IR HUVEC + vehicle (pg/mL)	IR HUVEC + LPS (pg/mL)	1 vs 2	3 vs 4	1 vs 3	2 vs 4
sCD40L	3.25 ± 0.49	5.41 ± 0.93	62.67 ± 10.83	105.33 ± 7.09	**	***	***	***
Eotaxin	1.55 ± 0.30	2.29 ± 0.48	32.83 ± 5.65	51.61 ± 4.53	*	**	***	***
FLT-3L	0.86 ± 0.03	0.92 ± 0.06	17.30 ± 2.61	24.12 ± 3.06	NS	*	***	***
Fractalkine	3.96 ± 5.02	14.46 ± 2.44	226.23 ± 34.29	274.62 ± 49.88	**	NS	***	***
G-CSF	125.68 ± 1.94	998.16 ± 153.67	25472.97 ± 4318.10	39264.89 ± 5329.63	***	**	***	***
GM-CSF	14.62 ± 0.75	108.49 ± 13.78	1843.58 ± 291.68	5074.77 ± 1423.94	***	**	***	***
GROα	683.37 ± 36.05	1689.77 ± 408.61	13823.24 ± 1849.17	21218.77 ± 4531.09	**	*	***	***
IFN-α2	3.91 ± 0.42	4.88 ± 0.46	58.55 ± 10.43	81.45 ± 16.95	*	0.06	***	***
IFNγ	0.28 ± 0.03	0.44 ± 0.05	5.53 ± 0.94	8.13 ± 1.46	**	*	***	***
IL1α	1.66 ± 0.05	3.44 ± 0.16	18.74 ± 3.79	41.19 ± 9.42	***	**	***	***
IL1β	0.82 ± 0.58	1.75 ± 0.43	27.35 ± 6.40	36.50 ± 10.84	*	NS	***	***
IL1RA	0.34 ± 0.02	0.56 ± 0.03	6.51 ± 1.24	9.07 ± 0.99	***	*	***	***
IL2	0.04 ± 0.04	0.17 ± 0.02	2.48 ± 0.52	2.77 ± 0.37	***	NS	***	***
IL3	0.03 ± 0.05	0.01 ± 0.01	0.64 ± 0.71	1.58 ± 0.60	NS	NS	NS	**
IL4	0.11 ± 0.02	0.18 ± 0.01	1.68 ± 0.15	2.23 ± 0.36	***	*	***	***
IL5	0.04 ± 0.01	0.05 ± 0.01	0.65 ± 0.08	0.98 ± 0.21	NS	*	***	***
IL6	620.70 ± 111.76	3370.38 ± 531.38	36667.56 ± 5926.87	54897.55 ± 10508.26	***	*	***	***
IL8	655.64 ± 99.89	2491.95 ± 412.60	52105.49 ± 1449.75	74359.67 ± 21676.26	***	0.08	***	***
IL9	1.29 ± 0.07	2.08 ± 0.05	23.26 ± 2.66	31.64 ± 3.07	***	**	***	***
IL10	0.36 ± 0.03	0.74 ± 0.09	8.92 ± 1.30	13.44 ± 1.22	***	**	***	***
IL12p40	3.45 ± 0.47	5.61 ± 0.42	64.81 ± 1.01	94.14 ± 12.44	***	**	***	***
IL12p70	0.02 ± 0.04	0.18 ± 0.17	1.09 ± 0.75	1.47 ± 1.29	NS	NS	*	0.09
IL13	1.64 ± 1.00	2.48 ± 0.64	35.57 ± 7.20	41.18 ± 11.60	NS	NS	***	***
IL15	2.35 ± 0.20	3.23 ± 0.12	70.19 ± 9.16	114.07 ± 20.15	***	**	***	***
IL17A	0.14 ± 0.08	0.63 ± 0.12	7.95 ± 0.38	8.55 ± 1.74	***	NS	***	***
IL17E/IL25	3.18 ± 0.64	7.55 ± 0.45	116.91 ± 14.03	169.48 ± 20.66	***	**	***	***
IL17F	0.47 ± 0.38	1.16 ± 0.23	22.10 ± 2.85	26.54 ± 4.69	*	NS	***	***
IL18	0.11 ± 0.03	0.19 ± 0.02	1.94 ± 0.25	2.98 ± 0.32	**	**	***	***
IL22	0.00 ± 0.00	18.17 ± 1.17	265.59 ± 50.25	400.75 ± 33.91	***	**	***	***
IL27	12.96 ± 8.76	14.82 ± 3.10	356.25 ± 46.48	394.11 ± 63.50	NS	NS	***	***
IP10	23.06 ± 3.51	136.26 ± 14.19	29473.26 ± 14267.88	29360.89 ± 11899.07	***	NS	**	**
MCP3	22.41 ± 0.64	72.38 ± 9.53	186.56 ± 27.39	305.38 ± 54.25	***	**	***	***
MCSF	34.67 ± 0.93	48.74 ± 2.95	615.57 ± 89.70	1044.27 ± 166.75	***	**	***	***
MDC	0.01 ± 0.01	0.05 ± 0.02	0.73 ± 0.23	1.07 ± 0.26	*	0.08	***	***
MIG/CXCL9	2.70 ± 0.24	6.25 ± 0.57	252.02 ± 55.18	378.85 ± 75.79	***	*	***	***
MIP-1β	0.00 ± 0.00	0.00 ± 0.00	26.05 ± 7.52	30.06 ± 6.11	NS	NS	***	***
PDGF-AA	430.88 ± 14.86	440.39 ± 58.44	754 ± 114.06	1142.28 ± 254.60	NS	*	***	***
PDGF-AB/BB	645.98 ± 11.48	758.43 ± 86.22	4730.46 ± 718.48	9212.95 ± 1718.97	*	**	***	***
RANTES	13.45 ± 1.88	21.75 ± 1.33	9275.92 ± 2226.48	17334.21 ± 16977.73	***	NS	***	0.08
TGFα	0.83 ± 0.09	1.32 ± 0.23	130.71 ± 25.25	223.88 ± 45.16	**	*	***	***
TNFα	3.39 ± 0.21	5.68 ± 0.48	130.71 ± 25.25	223.88 ± 45.16	**	*	***	***
TNFβ	1.88 ± 0.15	3.54 ± 0.27	49.75 ± 3.38	69.75 ± 11.17	***	*	***	***
Eotaxin-2	0.27 ± 0.11	0.65 ± 0.15	2.67 ± 1.45	4.78 ± 2.80	**	NS	*	*
MCP-2	3.98 ± 0.41	6.13 ± 0.58	2025.50 ± 320.31	4026.44 ± 427.57	***	***	***	***
BCA-1	0.31 ± 0.03	0.30 ± 0.04	2.98 ± 0.86	3.65 ± 0.71	NS	NS	***	***
I-309	0.42 ± 0.02	1.10 ± 0.11	2.19 ± 0.35	2.73 ± 0.49	***	NS	***	***
IL16	2.18 ± 0.70	2.20 ± 1.60	12.70 ± 21.47	22.57 ± 22.25	NS	NS	NS	NS
TARC	0.05 ± 0.04	0.07 ± 0.04	0.66 ± 0.61	0.91 ± 0.54	NS	NS	0.09	*

SCF	1.07 ± 0.35	1.19 ± 0.18	55.23 ± 10.57	113.49 ± 16.47	NS	**	***	***
IL33	1.67 ± 0.28	1.61 ± 0.50	15.92 ± 6.71	16.80 ± 4.49	NS	NS	**	***
IL21	0.74 ± 0.31	0.66 ± 0.16	7.62 ± 1.68	8.50 ± 3.16	NS	NS	***	**
IL23	20.58 ± 15.29	13.03 ± 6.14	182.15 ± 70.78	66.92 ± 65.34	NS	0.05	**	NS
TRAIL	2.42 ± 0.15	2.93 ± 0.41	71.80 ± 17.18	85.64 ± 10.35	0.06	NS	***	***
SDF-1α+β	0.00 ± 0.00	19.83 ± 24.89	232.60 ± 280.75	430.73 ± 290.46	NS	NS	NS	*
ENA-78	55.09 ± 5.29	407.35 ± 43.44	9255.07 ± 158.57	17449.80 ± 3021.68	***	**	***	***

(n = 4; mean ± SD; * p<0.05 ** p<0.01 *** p<0.001).

Supplementary Table 2. List of primers used for qPCR.

Gene	Forward primer	Reverse primer
GAPDH	GTCTCCTCTGACTTCAACAGCG	ACCACCCTGTTGCTGTAGCCAA
IL6	AGACAGCCACTCACCTCTTCAG	TTCTGCCAGTGCCTCTTTGCTG
IL1β	CCACAGACCTTCCAGGAGAATG	GTGCAGTTCAGTGATCGTACAGG
TNFα	CTCTTCTGCCTGCTGCACTTTG	ATGGGCTACAGGCTTGTCACTC
CCL2	GTCTCTGCCGCCCTTCTGT	TTGCATCTGGCTGAGCGAG
CCL5	CCTGCTGCTTTGCCTACATTGC	ACACACTTGGCGGTTCTTTCGG
CXCL1	AGGGAATTCACCCCAAGAAC	ACTATGGGGGATGCAGGATT
CXCL10	GGTGAGAAGAGATGTCTGAATCC	GTCCATCCTTGGAAGCACTGCA
p16	CTCGTGCTGATGCTACTGAGGA	GGTCGGCGCAGTTGGGCTCC
p21	AGGTGGACCTGGAGACTCTCAG	TCCTCTTGGAGAAGATCAGCCG

Supplementary Table 3. List of antibodies used for western-blot.

Protein	Dilution	Vendor	Cat. #
β-Actin	1:1000 in 5% milk	Cell Signaling Technologies, Danvers, MA, USA	4970S
GAPDH	1:1000 in 5% milk	Cell Signaling Technologies, Danvers, MA, USA	97166S
p-p38 MAPK	1:1000 in 5% milk	Cell Signaling Technologies, Danvers, MA, USA	4511S
p38 MAPK	1:1000 in 5% milk	Cell Signaling Technologies, Danvers, MA, USA	8690S
NF-κB p65	1:1000 in 5% milk	BD Biosciences, San Jose, CA, USA	610869
TLR4	1:200 in 5% milk	Santa Cruz Biotechnology, Inc., Dallas, TX, USA	sc-293072
IL1R1	1:200 in 5% milk	Santa Cruz Biotechnology, Inc., Dallas, TX, USA	sc-393998
IL1R2	1:200 in 5% milk	Santa Cruz Biotechnology, Inc., Dallas, TX, USA	sc-376247
TNFR1	1:200 in 5% milk	Santa Cruz Biotechnology, Inc., Dallas, TX, USA	sc-8436
Histone H3	1:1000 in 5% milk	Cell Signaling Technologies, Danvers, MA, USA	4499S
Anti-Mouse IgG (HRP-Linked)	1:3000 in 5% milk	Cell Signaling Technologies, Danvers, MA, USA	7076S
Anti-Rabbit IgG (HRP-Linked)	1:3000 in 5% milk	Cell Signaling Technologies, Danvers, MA, USA	7074S
